# Mechanisms of chromosomal rearrangement in the human genome

**DOI:** 10.1186/1471-2164-11-S1-S1

**Published:** 2010-02-10

**Authors:** Albert G Tsai, Michael R Lieber

**Affiliations:** 1 USC Norris Comprehensive Cancer Ctr., Rm. 5428 Departments of Pathology, Biochemistry & Molecular Biology, Molecular Microbiology & Immunology, and of Biological Sciences (Section of Molecular & Computational Biology), University of Southern California 1441 Eastlake Ave., MC9176 Los Angeles, CA 90089-9176, USA

## Abstract

Many human cancers are associated with characteristic chromosomal rearrangements, especially hematopoietic cancers such as leukemias and lymphomas. The first and most critical step in the rearrangement process is the induction of two DNA double-strand breaks (DSB). In all cases, at least one of the two DSBs is generated by a pathologic process, such as (1) randomly-positioned breaks due to ionizing radiation, free radical oxidative damage, or spontaneous hydrolysis; (2) breaks associated with topoisomerase inhibitor treatment; or (3) breaks at direct or inverted repeat sequences, mediated by unidentified strand breakage mechanisms. In lymphoid cells, one of the two requisite DSBs is often physiologic, the result of V(D)J recombination or class switch recombination (CSR) at the lymphoid antigen receptor loci. The RAG complex, which causes the DSBs in V(D)J recombination, can cause (4) sequence-specific, pathologic DSBs at sites that fit the consensus of their normal V(D)J recombination signal targets; or (5) structure-specific, pathologic DSBs at regions of single- to double-strand transition. CSR occurs specifically in the B-cell lineage, and requires (6) activation-induced cytidine deaminase (AID) action at sites of single-stranded DNA, which may occur pathologically outside of the normal target loci of class switch recombination regions and somatic hypermutation (SHM) zones. Recent work proposes a seventh mechanism: the sequential action of AID and the RAG complex at CpG sites provides a coherent model for the pathologic DSBs at some of the most common sites of translocation in human lymphoma – the bcl-2 gene in follicular lymphoma and diffuse large B-cell lymphoma, and the bcl-1 gene in mantle cell lymphoma.

## Background

Somatic cell chromosomal rearrangements are either physiologic events [V(D)J recombination or class switch recombination (CSR)] exclusive to lymphoid cells, or they are pathologic events, often found in neoplasia. This review focuses primarily on these somatic cell types. Constitutional rearrangements which arise during gametogenesis and the first nuclear divisions of the fertilzed egg, and rearrangements which occur during evolution and population selection are beyond the scope of this review, but similar principles of breakage and rejoining apply, particularly the rejoining of double-strand breaks by nonhomologous DNA end joining (NHEJ).

**Figure 1:**
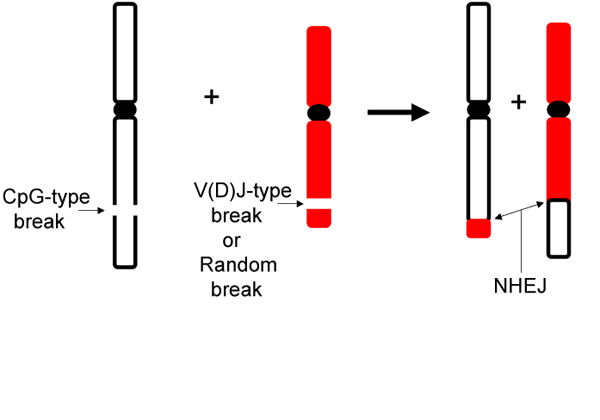
**Features of some neoplastic chromosomal translocations.** Chromosomal translocations in somatic cells are most often encountered in the context of neoplasia. Constitutional translocations arise during gametogenesis or early divisions of the fertilized egg. It is useful to dissect the chromosomal translocation process into two phases: the causes of the breaks and then the rejoining of the four broken ends. [Most chromosomal translocations involve two DSBs, though some can occur during DNA replication (see text).]  In neoplastic translocations, the causes of the two DSBs can be different. In this example, one chromosome break is of the CpG-type (see text), and the other can be a V(D)J-type or a random DSB.  In addition, breaks can be of the class switch type (CSR-type) or somatic hypermutation type (SHM-type). The V(D)J-type, CSR-type, SHM-type, and CpG-type are limited to lymphoid cells, and further limited by stage of differentiation (see text).  Random breaks are thought to be due to oxidative free radicals (reactive oxygen species), ionizing radiation (IR), or topoisomerase failures. The joining of the four DNA ends is done by nonhomologous DNA end joining (NHEJ) in most cases.  The causes of the breaks all generate heterogeniety at the break site, and NHEJ creates additional heterogeneity at the joining site. Hence, even when both translocation junctions are sequenced, one can only trace the site or boundary of the original breaks to a zone of at least several nucleotides (often longer, such as 10-20 bp). Less commonly, no nucleotides are lost from either DSB site, and this permits determination of the specific phosphodiester bonds at which the original DSBs occurred. This proved useful for defining the CpG-type break (see text).  Some of the specific translocations discussed here have been previously diagrammed in more detail [26-28]. The normal V(D)J and class switch recombination processes have been previously diagrammed [5-9] .

All cancers begin with somatic cell mutations. Chromosomal rearrangements are important types of mutations initiated by two double-strand DNA breaks (DSBs), resulting in four DNA ends (Figure 1). The four DNA ends can be rejoined in a new configuration as a translocation. Translocations are found at high frequencies in many malignancies, especially in the hematopoietic system, but are insufficient to drive carcinogenesis. Thus, they appear to be critical initiating events in such cancers, after which a variety of secondary and apparently less consistent mutations are required to complete the process.

Translocations occur in two steps: a cutting step and a joining step.  The joining step is usually done by the major repair pathway for double-strand DNA breaks, NHEJ.  NHEJ joins the ends of most DSBs in most eukaryotic cells, and it can join any two incompatible or compatible DNA ends, even though they usually have no homology. (In rare patients with mutations in NHEJ or in mouse models lacking all NHEJ factors, other enzymes can substitute for the missing factors, and this is often called alternative NHEJ [1-4].) The other major pathway is called homologous recombination, which is largely restricted to late S and G2 of the cell cycle and requires hundreds of base pairs of homology. The natural causes of DSB formation – the cutting mechanisms – are often more difficult to determine and are the focus of this review. As with somatic cell rearrangements, one can organize DSBs into those that are pathologic (maladaptive) and those that are physiologic (programmed or adaptive). Physiologic DSBs participate in physiologic rearrangements whereas pathologic DSBs by definition, do not. In the majority of pathologic chromosomal rearrangements, one of the two requisite DSBs is pathologic, and the other is physiologic (occurs during the course of a physiologic gene rearrangement process).

## Physiologic causes of double-strand DNA breaks

In the somatic cells of multicellular eukaryotes, physiologic DSBs only occur in the lymphoid cells of the vertebrate immune system as a way of generating a diverse array of receptors for binding to antigens of invading organisms.

### V(D)J recombination

B-cells and T-cells bind and recognize foreign antigens using the variable domains of B-cell receptors (immunoglobulins, or Ig) and T-cell receptors (TCR), respectively. Igs and TCRs are encoded by the Ig and TCR gene loci, which are organized into a set of V gene segments, followed sometimes by a set of D gene segments, followed by a set of J gene segments. Using the process of V(D)J recombination, early B cells (pro-B and pre-B cells residing in the bone marrow) and early T cells (pro-T and pro-T residing in the thymus) select one V, one D, and one J segment and join them together into the antigen receptor variable domain exon, deleting the intervening segments [5-9].

These early lymphoid cells express the RAG1 and RAG2 proteins, which combine with the constitutively-expressed HMGB1 or 2 to form the RAG complex. The RAG complex binds at special sequences termed recombination signal sequences (RSS) located directly adjacent to the V, D, or J segments. RSS are composed of a heptamer of consensus CACAGTG, and a nonamer of consensus ACAAAAACC, with an intervening spacer of 12 or 23 bp. A 12 bp-spaced RSS is termed a 12-RSS and a 23 bp-spaced RSS is termed a 23-RSS. After binding, the RAG complex makes a nick 5’ of the RSS heptamer sequence. This results in a single-strand break with a 5’ phosphate and a 3’ hydroxyl (OH). One 12-RSS and one 23-RSS must synapse, or bind the same RAG complex, before the complex can progress to the next step, which is use of the 3' OH to attack the opposite DNA strand, creating a hairpin DNA end at the terminus of the V, D or J segment, and a blunt DNA end containing the RSS.

Once the hairpins are generated, enzymes of the NHEJ pathway open the hairpins and join the resulting ends [10,11]. As a result, the requisite antigen receptor gene segments are assembled next to one another, forming a functional antigen receptor variable domain exon.

In B and T cell development, V(D)J recombination occurs not only in the earliest lymphoid precursors, but also in later stages of B and T cell development in secondary V(D)J recombination events called receptor editing or receptor revision. (Recombination failures that lead to translocations can occur at any point [1-4].)

### Class switch recombination

After V(D)J recombination, B cells, but not T-cells, can later undergo a second type of recombination event called class switch recombination (CSR), which changes the type of immunoglobulin they produce from IgM and IgD to IgG, IgA, or IgE. This change in the constant portion of the Ig heavy chain enhances phagocytosis of antibody-bound antigens and allows antibodies to penetrate extravascular regions of the body, such as epithelial surfaces in the lungs, gastrointestinal tract, and genitourinary system.

Downstream of the Ig heavy chain J segments is a set of constant domains, each preceded by a several kilobases-long CSR target region termed a class switch sequence [12,13]. Following successful V(D)J recombination, Ig heavy chain transcription runs through the variable domain, the IgM switch sequence, and the IgM and IgD constant regions. Alternative splicing determines production of IgM or IgD. Subsequently, B-cells can be activated by cytokine stimulation, which drives transcription through the IgG, IgA, or IgE switch regions. Different cytokines drive transcription through different switch regions. The RNA generated at switch regions is very G-rich, having a tendency to thread-back onto the template DNA strand from which they were transcribed, and causing formation of what is called an R-loop structure [14,15]. In an R-loop, the RNA is hybridized to its template DNA strand, with the nontemplate DNA strand left single-stranded.

CSR requires a protein called activation-induced deaminase (AID) [16], predominantly expressed in B cells located in the germinal centers of the lymph nodes, spleen, and Peyer's patches.  AID is a cytidine deaminase that converts cytosine (C) to uracil (U) or 5-methylcytosine (5-meC) to thymine (T). Importantly, AID only functions on single-stranded DNA (ssDNA) and is therefore a structure-specific deaminase [17-19].  AID generates Us in the nontemplate strand ssDNA at switch regions, which are excised by uracil DNA glycosylase (UDG), leaving abasic sites [20].  Apurinic/apyrimidinic endonuclease (APE) nicks the phosphodiester bond on the 5' side of any abasic site. Single-stranded regions occur on the template strand at the edges of the R-loop, where the RNA ceases to hybridize. Nicking on the nontemplate strand and on the template strand thus results in a DSB. We call these types of DSBs CSR-type. This term is useful for some translocations that require AID, but where the break is at a genomic site other than the Ig class switch regions. Such locations could be called off-target, and an example is the c-myc translocation in Burkitt lymphoma [3,4].

AID also functions in somatic hypermutation (SHM) at Ig variable domains of the light and heavy chains, causing point mutations, some of which increase the affinity of the Ig for its antigen [20]. It is not yet clear how single-strandedness arises at the variable domains to permit AID to function in SHM. Transcription alone appears to be insufficient, as not all transcribed sequences undergo SHM.

## Pathologic causes of double-strand DNA breaks

Pathologic DSBs are arbitrarily defined as DSBs which serve no physiologic purpose and may lead to cell dysfunction.

### Random DNA breaks due to ionizing radiation or oxidative free radicals

In many chromosomal rearrangements, the DSBs at one or both genes appear to be located randomly within large regions of many kilobases. Random positioning and the apparent lack of sequence propensity are suggestive of sequence-nonspecific DSB mechanisms such as oxidative free radicals, ionizing radiation, or less commonly, spontaneous DNA backbone hydrolysis.

About half of the natural ionizing radiation of the environment originates from natural heavy metals of the earth, such as uranium, thorium, and even potassium. The other half of the ionizing radiation emanates from cosmic radiation that is not entirely blocked by the atmosphere. In total, about 3 x 10^8^ ionizing radiation particles pass through each of us every hour [10], producing hydroxyl free radicals from water in their wake.  This tract of hydroxyl free radicals causes cluster damage on DNA, thereby breaking both DNA strands.

About 0.1% of the oxygen that we breathe is converted to free radicals [21]. This generates 3 x10^22^ free radicals per hour within each of us, and these damaging free radicals are distributed across the 10^14^ cells of the human body.  Free radicals cause predominantly single-strand DNA damage, but two nearby such events can result in a DSB.

### RAG action at cryptic RSS sites at off-target locations in a sequence-specific manner: V(D)J-type breaks

The RSS heptamer/nonamer consensus sequence is by no means unique to the Ig and TCR loci, and the RAG complex can cut at sites which differ substantially from the 16 bp consensus [22]. The minimal motif for RAG nicking is only CAC. Thus the RAG complex can act at RSS-like non-antigen receptor locus sites, termed cryptic RSS (cRSS). This occurs in many of the rearrangements observed in human T-cell acute lymphoblastic lymphoma [23-25].  In these cases, instead of the RAG complex pairing a 12-RSS with a 23-RSS, a 12-RSS pairs with a 23-cRSS or a 23-RSS pairs with a 12-cRSS. We call these breaks V(D)J-type breaks because they are occurring via the same mechanism as normal V(D)J recombination, regardless of the fact that one of the sites is outside of the usual antigen receptor loci (that is, it is off-target). 

### RAG action at DNA bubble structures and other regions of heterology in a structure-specific manner

In addition to its sequence-specific mode of cutting, the RAG complex can also nick in a structure-specific manner at sites of transition from dsDNA to ssDNA, such as occurs at the edges of bubble DNA structures or even single-base mismatches [26-28]. Such activity by the RAG complex may have arisen because the RAG complex is accustomed to creating hairpin structures, which involves substantial DNA distortion. Hence, any region of mismatch or slippage is a potential target for nicking by the RAG complex in lymphoid cells.

### RAG-mediated transposition as a mechanism for chromosomal rearrangement

From 1998 to 2007, several laboratories proposed that the RAG complex might insert the blunt RSS-containing ends from V(D)J recombination, termed signal ends, into new locations in the genome. This is called RAG transposition, and occurs at a low level using a truncated form of the RAG proteins called core RAGs (reviewed in [7]). However, efforts to find RAG transposition events in vivo showed that these were much less common than random integration of DNA [29]. Finally, there are no examples of human lymphoid malignancies (or any other type of malignancy) where the genome was altered by a RAG transpositional insertion of signal ends (or any other apparent variant of such a transposition).

### AID action at off-target locations

As mentioned in the above discussion of class switch recombination, AID can convert C to U or methyl C or T at any region of ssDNA. This appears to occur not only at the switch sequences and variable domains of the Ig loci, but also at some pathologic locations, such as some oncogenes like c-myc [3,4]. When targeted by AID, these regions may sustain point mutations or DSBs [30]. AID action at the IgH switch region during CSR and independent AID action at the c-myc gene to create a DSB are thought to be the basis of the two initiating DSBs in both mouse and human c-myc translocations [1-4]. One could regard breaks of this type as CSR-type breaks (as mentioned above in the discussion of class switch recombination) or SHM-type breaks, where SHM refers to AID initiated events of the type similar to what normally occurs in somatic hypermutation.

### Putative combined action of AID and RAGs at CpG sites: CpG-type breaks

Recently, we reported that DSBs at certain loci in pro-B/pre-B stage translocations – the bcl-2 from t(14;18), the bcl-1 from t(11;14), and E2A from t(1;19) – have a strong propensity to occur at the dinucleotide sequence CpG.

The bcl-2 translocation is the most common translocation in cancer, occurring in >90% of follicular lymphomas and a third of diffuse large cell lymphomas. Fifty percent of the breaks at the bcl-2 gene occur within the major breakpoint region (MBR), which is a 175 bp hotspot in the 3' most exon in the region encoding the 3'UTR. Two less-frequently used hotspots are located 18 and 29 kb further distal to the bcl-2 gene, the 105 bp bcl-2 intermediate cluster region (icr), and the 561 bp bcl-2 minor cluster region (mcr), respectively. Any of the CpG sites within any of these three bcl-2 translocation zones can be a target for a DSB [28]. Thirteen percent of bcl-2 translocation breaks are located in the icr, and 5% in the mcr.

The use of CpGs applies also to the bcl-1 major translocation cluster, which is the location involved in the t(11;14) translocation. The bcl-1 translocation occurs in almost all mantle cell lymphomas, with 30% of the breaks occurring at the 150 bp bcl-1 major translocation cluster (MTC).

CpG-type breaks also occur in a third lymphoid malignancy, the t(1;19) in a small percentage of pre-B ALLs, a translocation which occurs between the Pbx1 gene and the E2A gene. The breaks at the E2A gene occur in a zone of only 23 bp, and these DSBs are also significantly clustered around CpG sites [28]. All three translocations involving the bcl-2, bcl-1 and E2A occur at the pro-B/pre-B stage of B-cell development.

 The bcl-2 MBR is reactive with a chemical probe for single-strandedness called bisulfite [27]. Like the bcl-2 MBR, this bcl-1 MTC is relatively small (150 bp) and features a similar reactivity to bisulfite [31]. These highly bisulfite reactive zones are rich in runs of Cs. Based on circular dichroism, X-ray crystallography, NMR, and chemical probing, such runs of Cs tend to adopt a DNA structure that is intermediate between B-form DNA and A-form DNA, termed B/A-intermediate [31]. The B/A-intermediate structure has more rapid opening kinetics, perhaps accounting for part of the observed increase in bisulfite reactivity. Such unusual DNA regions may be more prone to slippage events, perhaps induced by DNA replication or transcription. This may then account for their vulnerability in minichromosomal recombination assays [27].

The Cs of the CpGs within or directly adjacent to these B/A-intermediate zones are at increased risk of undergoing deamination [28]. This deamination does not apply to all Cs in the region, but only the Cs that are within CpG sites. The only distinctive feature about such Cs within CpGs is that they can be methylated by DNA methyltransferase.  When regular Cs deaminate, they become U, resulting in a U:G mismatch.  But when methyl Cs deaminate, they become T, resulting in a T:G mismatch. The repair of U:G mismatches is very efficient, but the repair of T:G mismatches is not efficient. In fact, T:G mismatch repair is so inefficient, it accounts for about half of the point mutations at the p53 gene across a wide range of human cancers. These T:G mismatch sites are always at CpG sites.

What causes the break at these T:G mismatch sites?  Interestingly, this deamination at these lymphoid translocation hotspots appears to occur at the pre-B stage of differentiation.  This is the stage of B cell development when D to J recombination is occurring most vigorously. Since the bcl-2 and bcl-1 translocations occur at this stage, this seems likely to be the stage of the translocation.  We have shown that the RAG complex can cause a DSB at sites of small bubble structures, and even single base pair mismatches. (As mentioned above, this action by the RAG complex reflects its structure-specific nuclease activity, perhaps a feature that reflects the structure-specific actions by the RAG complex during the hairpin formation step of V(D)J recombination.) Therefore, we have proposed that the RAG complex makes the DSBs at the sites of T:G mismatch [28].

If the RAG complex causes the DSBs at CpG sites, then why do such CpG-type breaks not occur in pre-T cells, which also express the RAG enzyme complex? The B cell lineage expresses a cytidine deaminase for class switch recombination and somatic hypermutation. As mentioned above, this enzyme is called activation-induced deaminase (AID). AID is expressed in B cells but not other somatic cells. AID is most highly expressed in B cells when they are in the germinal centers. However, a low level of AID expression has been described in pre-B cells [32-34]. Moreover, B cells just leaving the bone marrow, called transitional B cells, also are thought to express AID [1].  Therefore, there is a period of time when B cells are completing V(D)J recombination and beginning to express AID when both AID and the RAG complex are present in the B cells. AID has been shown to be capable of deaminating methyl C to T.  Therefore, we propose that AID is likely responsible for the mutation of meC to T at CpG sites in early B cells. The resulting T:G mismatch is then cut by the RAG complex, resulting in a DSB. This model explains three peaks of translocation located within the bcl-2 MBR, all of which are centered at CpG sites [28].

### Other causes of pathologic DSBs of unknown mechanism

Certain translocations are heavily associated with type II topoisiomerase inhibitor therapy [35]. After such therapy, some patients develop secondary malignancies with these characteristic translocations. Topoisomerases in general make single- or double-strand breaks in order to wind or unwind DNA, thus they have a nuclease activity as part of their function. After winding or unwinding the DNA, they normally reseal the break(s). It has been proposed that interruption or prevention of resealing may result in stable breaks seen in chromosomal rearrangements [36,37].

Some DSBs arise at sites nearby direct or inverted DNA repeats. Such repeats may give rise to slipped DNA structures containing regions of single-stranded DNA, which may be targets for cleavage. The best example of this is the constitutional translocation t(11;22)(q23;q11), which contains an AT-rich palindrome of several hundred bases, with potential for cruciform formation.

### Combination of multiple DSB mechanisms within a rearrangement

Given that two DSBs are required to generate a translocation, the two breaks are often not related to one another.  In the bcl-2 and bcl-1 translocations, for example, the break at the IgH locus is a V(D)J-type break generated by the *sequence*-specific action of the RAG complex during V(D)J recombination. (One could consider this to be a failure in the completion of the normal V(D)J recombination process [23-25].) The DSB at the bcl-2 or bcl-1 locus is a CpG-type break that has been proposed to be due to the sequential action of AID and the *structure*-specific nicking activity of the RAG complex [28].

Even within a given locus, there can be a wide range of DSB mechanisms. The SCL and LMO2 loci predominantly both sustain V(D)J-type DSBs, but one-third or more of the DSBs are incompatible with the sequence requirements for V(D)J-type DSBs, and these may be due to free radical damage, ionizing radiation, or topoisomerase failures. Different loci within a single cell are therefore prone to different types of DSB mechanisms.

### Replication-induced DSBs

During DNA replication, deletions can arise due to slippage of the synthesizing strand on the template strand. Chromosomal rearrangements that occur at specific hotspots, whether in cancer in somatic cells or during gametogenesis/initial developmental divisions as constitutional translocations, are called recurrent translocations that can be seen across many patients. Nonrecurrent translocations are those that occur at different locations from one patient to another but alter or inactivate a gene that causes a disease.  Unlike the recurrent translocations that we have discussed in cancer above, the mechanisms that cause the strand exchange in nonrecurrent translocations appear to involve template switching during replicative DNA synthesis. These template switches can occur at small regions of DNA sequence homology, such as 5 bp. This template switching has been called microhomology-mediated breakage-induced replication (MMBIR) or Fork Stalling and Template Switching (FoSTeS). For nonrecurrent translocation junctions that involve several long stretches of sequence from regions of the genome that are normally separated from one another, multiple template switching events has been proposed as a mechanism [38,39].

## Biological factors and neoplastic chromosomal translocations

This review has not emphasized the neoplastic cellular proliferation advantage provided by these chromosomal rearrangements [40,41]. Rather, here we have focused on the factors that make some very focal (23 to 561 bp) DNA sequences particularly prone to repeated rearrangement events in a wide range of different patients, even though the translocations could have arisen within zones of 29 kb, as in the case of the bcl-2 gene or 100 kb, as in the bcl-1 translocation.

Recurrent rearrangements found in particular cancers are oncogenic, and by nature provide a growth advantage. Some such rearrangements result in the genesis of chimeric fusion transcripts between two genes, and such is the case for the t(1;19) translocation involving E2A and Pbx1 [42-47]. In these cases, the DSB could, in principle, occur across much of the length of a given intron within each gene so as to create the neoplastic fusion transcript. For the Pbx1 gene, the breaks occur diffusely in an intron in this way, and these DSBs are likely random and due to mechanisms such as free radical damage (i.e., ROS), ionizing radiation (IR), or topoisomerase failures. But the break at the E2A gene is focused to a 23 bp zone that is 150-fold tighter than the 3.2 kb intron in which it is located, and this is a CpG-type break site.

The same concepts apply to the t(9;22) translocation, the hallmark in chronic myeloid leukemia (CML) and also common in the t(9;22) ALLs in which the BCR gene and the ABL gene fuse and produce a p210 fusion transcript [48]. DSB formation at both the BCR gene and the ABL gene occur over broad translocation zones of more than 5.8 kb at the BCR gene and a much broader approximately 200 kb zone at the ABL gene [49-52]. Among the translocations that give rise to malignancy, the fusion gene minimally contains the first exon of BCR, which contains an oligomerization domain, and almost always contains exons 2 to 11 of ABL, which encode the tyrosine kinase. The 200 kb breakage zone at ABL contains translocation break sites distributed anywhere between alternative exons 1a and 1b. The 5.8 kb breakage zone at BCR is the major breakpoint cluster region (M-BCR) and encompasses a region containing exons 13 and 14. [A minor BCR, m-BCR (~130 kb in length), can give rise to a shorter p190 fusion gene and is located in intron 1.] Leukemia only arises from those fusion genes that can produce an mRNA encoding a functional protein: thus, only certain splice combinations produce a BCR/ABL protein. Therefore, it is not the increased breakage in the BCR that makes it a translocation zone; it is the lack of growth advantage outside of the zone which demarcates the boundaries of where the zone can be.

Again, the DSBs at both the BCR and ABL are likely due to random causes (ROS, IR, toposimerase failures).  Therefore, it is useful to distinguish between focal hotspots (i.e., high concentrations of breaks at small zones within the total breakage zone, usually <200 bp), as in the bcl-2, bcl-1 or E2A cases, and broad translocation zones that often span the length of an entire intron (usually kilobases, and sometimes tens of kilobases or more).

For translocations involving focal hotspots, there are two factors that make the translocation clinically apparent: the increased propensity for DSB and the growth advantage.  In contrast, for translocations that have no hotspot propensity, then only the growth advantage acts to bring the translocation to the level of clinically attention [28].

## Relevance to constitutional chromosomal rearrangements and to changes in a genome during evolution

We have focused here on neoplastic chromosomal rearrangements. As mentioned, the breakage and rejoining mechanisms and concepts may be relevant to constitutional translocations or changes in a genome during evolution. The most common constitutional chromosomal rearrangement is the t(11;22) in the Emanuel Syndrome [53]. In this case, inverted repeats result in cruciform formation, creating a DNA structure that is vulnerable to DNA enzymes that can act on various portions of the cruciform structure. Once broken, the DNA ends likely join by NHEJ. Hence, the concepts of DNA structural deviation, followed by inadvertent action of a physiologic DNA enzyme to cause the break and rejoining by NHEJ have more general relevance.

During evolution, some of the chromosomal rearrangements that arise during speciation are almost certain to share themes with those discussed here, including breakage at sites of DNA structural variation and joining by NHEJ. Replication-based mechanisms mentioned briefly here and discussed in detail elsewhere are also likely to be very important for major genomic rearrangements [38,39].

## List of abbreviations used

NHEJ: nonhomologous DNA end joining; RAG: recombination activating gene; AID: activation-induced deaminase; RAG complex: RAG1:RAG2:HMGB1 proteins in a complex; V(D)J: variable (diversity) joining, which refers to segments of DNA joined by the RAG1:RAG2:HMGGB1 complex to generate the variable domain exons of immunoglobulins and T-cell receptors; V(D)J recombination: the process of joining of V, D, and J coding segments together to form the variable domain exon; RSS: recombination signal sequence, which is also called the signal sequence. The RSS or signal sequence is the site where the RAG complex binds. The RSS or signal sequence consists of a heptamer (CACAGTG; this consensus sequence can vary at the right side from one signal to another) and a nonamer (ACAAAAACC; this consensus sequence varies quite a bit); Coding sequence: any sequence adjacent to an RSS; Coding end: the DNA end generated by RAG cleavage. This has a hairpin configuration until it the hairpin is opened by the Artemis:DNA-PKcs complex; Signal end: the DNA end at the RSS side generated by RAG cleavage. This has a 5'P and 3'OH; CSR: class switch recombination; Class switch sequences or regions: repetitive units of length 25 to 80 that compose the class switch recombination regions in mammalian B cells; ALL: acute lymphoblastic leukemia or lymphoma. The distinction of the leukemia or lymphoma for ALL is largely arbitrary. If the patient presents with extensive bone marrow and peripheral blood ALL cells, then the designation leukemia is preferred. If the patient presents with a mass lesion and <25% ALL cells in the bone marrow, the term lymphoma is preferred.

## Competing interests

The authors declare that they have no competing interests

## Authors' contributions

Both authors wrote all portions of the text.
